# Investigation and management of osteoporosis in aged trauma patients: a treatment algorithm adapted to the German guidelines for osteoporosis

**DOI:** 10.1186/s13018-017-0585-0

**Published:** 2017-06-08

**Authors:** Carl Neuerburg, Lena Mittlmeier, Ralf Schmidmaier, Christian Kammerlander, Wolfgang Böcker, Wolf Mutschler, Ulla Stumpf

**Affiliations:** 1 Department of General, Trauma and Reconstruction Surgery, Munich University Hospital LMU, Campus Großhadern, Marchioninistr. 15, 81377 Munich, Germany; 2 Department of Endocrinology, Medizinische Klinik und Poliklinik IV, Klinikum der Universität München, Munich, Germany; 3 Department of Trauma Surgery, Medical University, Innsbruck, Austria

**Keywords:** Fragility fractures, Hip fractures, Orthogeriatrics, Osteoporosis, Treatment algorithm

## Abstract

**Background:**

Osteoporosis-associated fractures are of increasing importance in trauma surgery. Systematic diagnostics and treatment of osteoporosis during a hospital stay, however, remain inadequate. Therefore, a specific algorithm for diagnosing and treating osteoporosis in trauma surgery patients was developed based on the DVO (German Osteology Society) guideline for osteoporosis from 2014.

**Methods:**

In a first step, the individuals’ age and risk profile for osteoporosis is identified considering specific fractures indicating osteoporosis and risk factors assessed by a specific questionnaire. In addition, physical activity, risk of falls, dietary habits and the individuals’ medication are considered. Basic osteoporosis laboratory tests, a bone densitometry by dual-energy X-ray absorptiometry (DXA) and, if needed, X-rays of the spine are carried out to identify prevalent vertebral body fractures.

**Results:**

Based on the treatment algorithm adapted to the new guidelines for osteoporosis in the majority of proximal femoral fractures, treatment of osteoporosis could already be indicated without prior DXA. In case of preexisting glucocorticoid therapy, a history of previous fractures or other risk factors according to the risk questionnaire, the threshold of treatment has to be adjusted given the table of *T*-scores.

**Conclusions:**

The treatment algorithm for diagnosing and treating osteoporosis in in-patient trauma surgery patients can help identify high-risk patients systematically and efficiently. As a result, osteoporosis-associated fractures or failure of osteosynthesis could be reduced, yet a prospective validation of the algorithm has to be completed.

## Background

Osteoporosis is a frequent underlying disease in elderly patients with fractures following low-energy trauma which is often identified too late or not at all, and therefore, trauma surgeons play a key role in the investigation and management of the disease.

Manifest osteoporosis leads to limited life expectancy and quality of life. Osteoporotic fractures are often accompanied by the loss of independence. The prevalence of osteoporosis in female patients >75 years old is estimated at 59.2% [1]. It is assumed that in the Federal Republic of Germany 6.3–7.8 million people suffer from osteoporosis [1, 2]. Up to 27% of the patients have already suffered a fracture. Patients who have had multiple fractures run an 85% risk of suffering another fracture within a year if not treated with drugs [3]. However, only 21% of osteoporosis patients in Germany receive guideline-oriented treatment [1]. A possible explanation for inadequate diagnostics and treatment of osteoporosis is certainly the increasing complexity of a guideline-oriented procedure. The DVO guideline creates transparency in view of the increasingly complex scientific evidence. Adapted to the current osteoporosis guideline of the DVO (Dachverband Osteologie = German Osteology Society) from 2014 [4], a diagnostics and treatment algorithm for treating inpatients in trauma surgery has been developed in order to eliminate the deficit in treatment. This algorithm has been developed to make use of existing scientific evidence pragmatically in trauma surgery on a daily basis. The aims of this algorithm are to improve on the one hand the deficit in diagnosing osteoporosis as an underlying disease in trauma patients and, on the other hand, to reduce the deficit in treatment of osteoporosis as a systemic skeletal disorder to reduce following osteoporotic fractures.

## Methods

### Identification of patients related to osteoporosis

#### Patients with fracture

In general, a distinction has to be made between patients with and without fractures typical of osteoporosis (thoracic and lumbar vertebral fractures, proximal femoral fractures, proximal humerus fractures and distal radius fractures) [5]. Additional clarification is advisable in female patients >50 and male patients >60 years old who have already suffered a fracture indicating osteoporosis. In a previous examination of trauma surgical patients with such a fracture, osteoporosis was established in 56.2% of women >50 and 59% of men >60 years old [6].

#### Patients without fracture

**Fig. 1:**
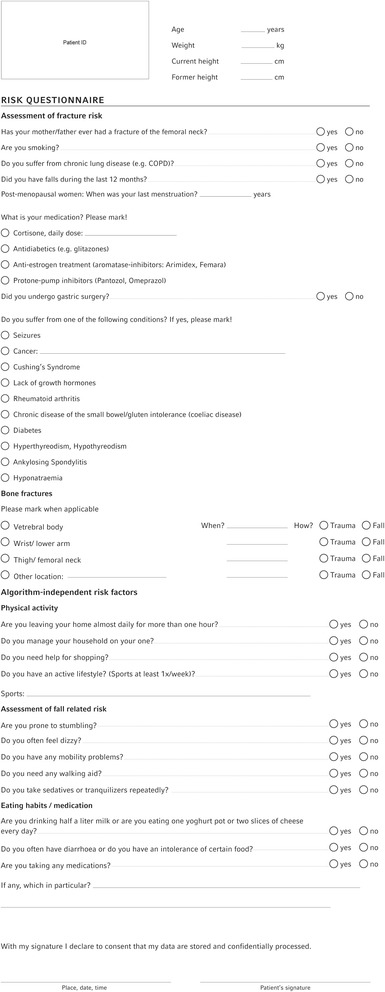
Osteoporosis risk questionnaire

It is always important to establish whether women >70 and men >80 years old are suffering from osteoporosis, whereas for younger patients, this only makes sense if there are specific risk factors (DVO guideline 2014). Specific risk questionnaires are a reliable and cost-effective method of determining the individual risk [7]. The individual risk of fractures can be determined reliably from the evidence-based risk factors. The FRAX and the Q-Fracture score, for example, are internationally accepted means for calculating osteoporosis-associated risks of fractures [8, 9]. The risk model of the DVO represents a further means for calculating the risk of fractures. We developed a risk questionnaire based on this (Fig. 1). The yes/no questions are clearly comprehensible for any patient and offer only one possible answer. Once one of the questions is answered by “yes”, the risk profile is assumed to be positive and it has to be established whether the patient is suffering from osteoporosis or not. In a later step of the algorithm, the risk profile is taken up again and will influence the therapeutic decision based on the *T*-score determined by dual-energy X-ray absorptiometry (DXA) (if necessary, the treatment threshold has to be raised by +0.5). In addition, individual physical activity, risk of falling, dietary habits and current medication will be included. In clinical practice, this enables the doctor in charge to decide individually how to diagnose and treat osteoporosis in a time-saving and cost-effective way.

### Diagnostics of patients related to osteoporosis

#### Evaluation of basic laboratory tests

**Fig. 2:**
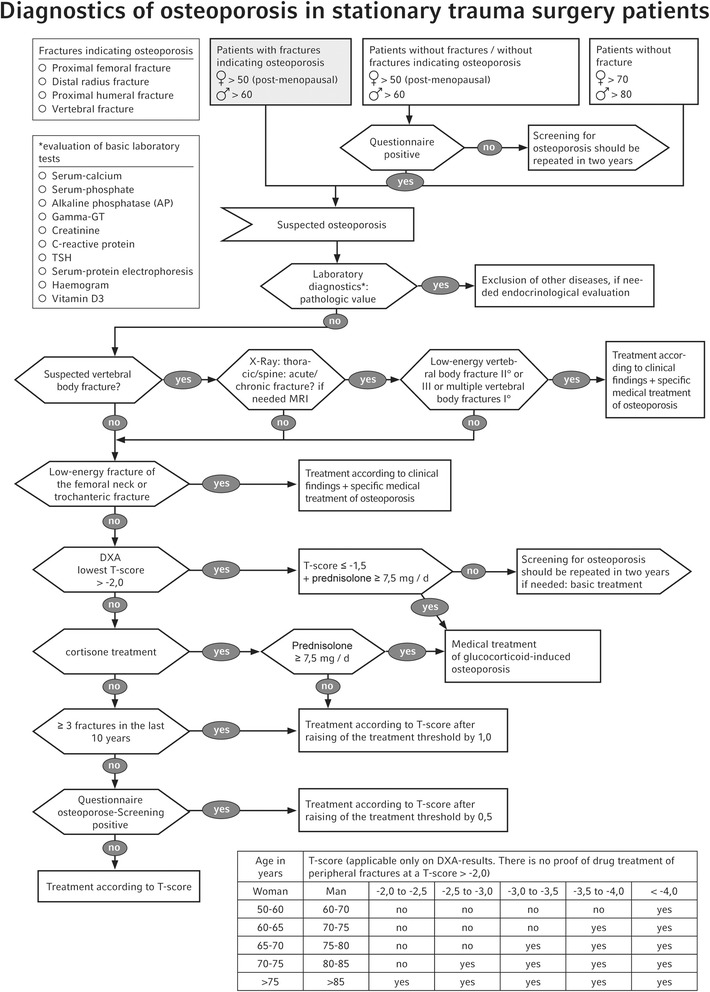
Osteoporosis diagnostic algorithm

The first step of the entire diagnostic process leading to the diagnosis of osteoporosis is the laboratory diagnostics (Fig. 2). As the decrease in bone density in terms of secondary osteoporosis may be caused by hitherto unknown co-morbidities or changes in the metabolism, it is important to establish the most important risk factors that can be identified in a laboratory [10]. In addition to that, basic laboratory tests contribute to checking important contraindications for medical treatment. Table 1 shows a list of important co-morbidities caused by specific changes in laboratory findings.

**Table 1:** List of most important causes related to changes in laboratory findings, according to the DVO guideline 2014 [4]

Laboratory parameter	Diagnostic interest
Serum-calcium	↑Primary hyperparathyroidism, tumour hypercalcaemia ↓e.g. secondary hyperparathyroidism, malabsorption, hypercalcaemia and hypocalcaemia as contraindications for several drugs against osteoporosis
Serum-phosphate	↑Renal insufficiency stage IV ↑Secondary renal hyperparathyroidism ↓Malabsorption
Serum-sodium (optional)	↓Greater risk of vertebral and non-vertebral fractures
Alkaline phosphatase (AP) (serum)	↑e.g. osteomalacia
Gamma-GT	For differential diagnosis of AP-increase caused by hepatitis, evidence for coeliac disease or alcohol abuse (risk of falling)
Creatinine-clearance	↓Renal osteopathy Severe renal insufficiency as contraindication for various drugs
ESR (erythrocyte sedimentation rate)/CRP (C-reactive protein)	↑Differential diagnosis for inflammatory causes of vertebral body deformities, inflammatory rheumatic diseases, multiple myeloma
full blood count	Evidence of inflammatory and malignant diseases or coeliac disease
Serum-protein electrophoresis	Evidence of monoclonal gammopathy or hypogammaglobulinaemia as evidence of MGUS or multiple myeloma; polyclonal hypergammglobulinaemia in systemic inflammatory diseases
TSH	<0.3 mU/L caused endogenously or by L-Thyroxine medication as a risk factor for fractures
If necessary, testosterone for men	Testosterone deficiency
If necessary, 25-hydroxy-vitamin D3 in individual cases	Vitamin D deficiency
If necessary, bone resorption parameter in individual cases (inconsistent data for men)	Fracture risk due to a high rate of bone re-formation

#### Indication of vertebral body fractures

Vertebral body fractures are a common sign of osteoporosis. Prevalent vertebral fractures are associated with high risk of impaired function and additional fractures. Due to static changes such as kyphosis and the shortening of the affected vertebral sections, they may cause numerous clinical symptoms and syndromes [11], like progressive kyphosis of the thoracic spine (“dowagers hump”), the loss of height >4–10 cm and typical skin folds appear that run down the back laterally to the flanks, referred as “fir tree phenomenon”. Often spinal compression fractures occur clinically asymptomatically and without previous trauma. They account for 11–15% of osteoporotic fractures [12]. As a result, the risk of further vertebral body fractures increases considerably [13]. Furthermore, degenerative changes in the vertebral column and vertebral fractures have a significant influence on bone densitometry by DXA, as such changes may lead to an incorrectly high bone densitometry [14, 15].

Since up to 75% of vertebral fractures do not come to clinical attention at the time of their occurrence, spine imaging with X-ray is required for their detection. For vertebral fracture assessment on standard lateral spine radiographs, the Genant semiquantitative method is used as the gold standard [16]. With this method, vertebral fractures can be graduated from 0 (normal) up to III (severe). Form and extent (loss of height) of the fracture were characterized. Multiple vertebral body fractures I° (Genant) or singular vertebral body fractures II–III° (Genant) in female patients >50 and male patients >60 years old need specific medical treatment, independent of the bone density measured by DXA according to the DVO guideline 2014. In this respect, the morphology of the vertebral column plays an important part in diagnosing and treating osteoporosis. Therefore, the synopsis of clinical signs of vertebral fractures and the severity of vertebral fractures according to Genant play a very important role in our algorithm to start immediately a specific treatment of osteoporosis.

#### Fractures of the femoral neck or pertrochanteric fractures

Proximal femoral fractures as femoral neck fractures and pertrochanteric fractures are diagnosed by radiography. For characterization of femoral neck fractures, the classification of Garden is frequently used. Pertrochanteric fractures are classified by the AO/OTA classification. As indicated in the osteoporosis algorithm, there is a specific link for “proximal femoral fractures”: according to the published draft of the DVO guideline 2014, the indication for specific medical treatment of osteoporosis was set for all patients with fractures of the femoral neck and a *T*-score <−2 as well as for those with a low-trauma pertrochanteric fracture (independent of the *T*-score). This indication for treatment of osteoporosis was reduced in the subsequently adopted version of the DVO guideline 2014 and replaced by the statement that treatment is generally indicated even without measuring bone density in typical radiological and/or clinical aspects of proximal femoral fractures [4]. In the authors’ opinion, however, the above mentioned femoral fractures have a significant relevance as a diagnostic criterion for the initiation of osteoporosis treatment, because of the high risk of mortality associated with these, the high risk of follow-up fractures and the influence on the quality of life connected with that [17].

This criterion was nevertheless taken up in the algorithm, because it helps to significantly facilitate the start of the treatment of osteoporosis in trauma surgical practice (Fig. 2). Osteoporosis treatment with drugs initiated after a proximal femoral fracture is accompanied by a significant reduction in new fractures and optimised survival [18]. The Working Group Geriatric Traumatology of the German Society for Trauma Surgery (AG Alterstraumatologie der Deutschen Gesellschaft für Unfallchirurgie) predicts that the number of proximal femoral fractures will rise dramatically by 351% by 2050 due to demographic development [19]. This estimate underlines the importance of ascertaining the diagnosis for osteoporosis mentioned above.

#### Bone densitometry

The definition of Osteoporosis by the World Health Organisation (WHO) is characterized by using a densitometric definition based on areal bone mineral density measured with DXA [20]. Due to this and solid data, low costs and a low level of exposure to radiation, bone densitometry by DXA is still used as the gold standard for analysing bone density [21]. A decrease in bone density raises the risk of an osteoporotic fracture [22]. Data for bone density in the area of the lumbar column (lumbar column body 1–4) and of the proximal femur collected during that process are compared with the data for bone density in a standard control group (i.e. *T*-score). Together with the age of the patient and the individual risk factors, this score forms the basis for the decision for a specific osteoporosis treatment. DXA as diagnostic part can be found in our algorithm at different points: important for a pragmatic approach is from our point of view that there are two important fractures (vertebral fractures and proximal femoral fractures) where the diagnosis osteoporosis is clear and a specific medication can be started without initial DXA as diagnostic part.

#### Risk factors relevant for treatment

A large number of risk factors have significant influence on the bone metabolism and the development of osteoporosis. In addition to the risk factors for the decision for a specific osteoporosis treatment already mentioned above, medical treatment with glucocorticoids should also be considered; the risk of suffering an osteoporotic vertebral body fracture during this treatment is significantly increased [23]. Therefore, specific medical treatment is already indicated when the *T*-score is ≤−1.5 and when treated daily with 7.5 mg prednisolone (see Fig. 2 for *T*-score chart).

Individual fracture anamnesis is also of considerable relevance for the risk of suffering further fractures [24]. According to the DVO risk calculation, this risk factor leads to a raising of the treatment threshold by means of the *T*-score by +1.0. Other risk factors may also lead to a raising of the treatment threshold by +0.5 and are therefore to be collected consistently using the risk questionnaire mentioned above (Fig. 1) in order to make an individual identification of osteoporosis according to the attached *T*-score chart (Fig. 2) possible.

### Treatment of osteoporosis

**Fig. 3:**
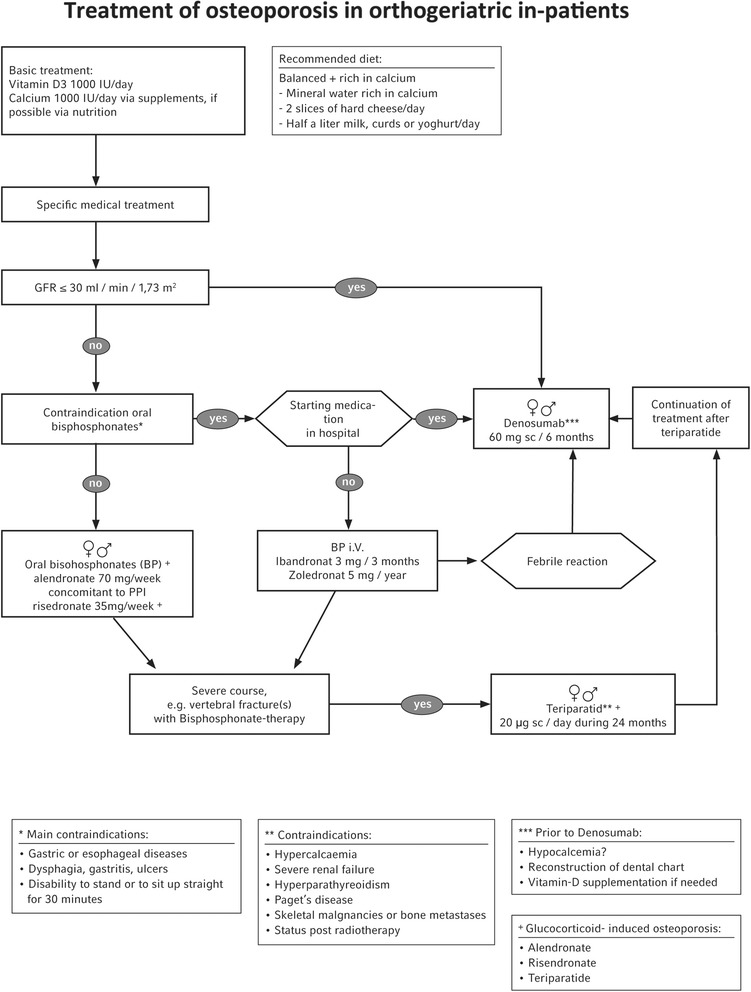
Osteoporosis treatment algorithm (adapted from Schray D et al. 2016 [51])

After having detected risk patients by means of our risk questionnaire and having diagnosed osteoporosis according to our algorithm, specific treatment of the osteoporosis is initiated (Fig. 3). A negative impact on the healing of fractures caused by anti-resorptive treatment by bisphosphonates cannot be proved [25, 26]. Ingrowth of prostheses (also cemented ones) [27, 28] is supported by anti-resorptive treatment. There are clinical studies that show that the osteoanabolic treatment of fractures with teriparatide has a positive influence on the healing process [29, 30].

If there are any questions concerning treatment strategies for complex osteological issues or for patients with a serious course of the disease, we have an osteological team constantly available for consultation by the colleagues on the ward in our clinic.

When choosing a drug for specific treatment of osteoporosis, the specific registration for the relevant indication and the lack of contraindications must be considered. The basis for this is the latest expert information.

#### Basic treatment

According to the guidelines of the DVO, the basic treatment stays the intake of 1000 mg calcium/day via nutrition, supplemented by 800–1000 IU vitamin D/day as a maintenance dose.

#### Vitamin D deficiency

A 25-hydroxy-vitamin D concentration in the serum <50 nmol/l or <20 ng/ml is accompanied by a moderately increased risk of proximal femoral fractures and non-vertebral fractures in elderly men and post-menopausal women [29–32]. The osteologically recommended range for adequate treatment is a serum level of 30–150 ng/ml (75–375 nmol/l) [33–36]. The synthesis of vitamin D3 in the skin under the impact of UV-light decreases with age, which is due to less exposure to sunlight and a reduction in the functioning of the skin [34, 37, 38]. Because there is a high proportion of elderly and very old people as patients in trauma surgery and geriatric traumatology and because it is by definition a question of manifest osteoporosis (with previous fracture), we generally recommend that the 25-hydroxy-vitamin D level in this group of patients be initially checked, in accordance with the DVO decision in individual cases. Studies show that only 7% of patients with a proximal femoral fracture have a sufficient 25-OH-vitamin D level.

If hypovitaminosis D (with secondary hyperparathyroidism) is proved, we recommend compensation with 20,000 IU Dekristol/week along with serum-calcium check-ups until the 25-OH-vitamin D level is in the desired area mentioned above (and the iPTH has normalised). High-dose treatment with a single administration of 50,000 IU vitamin D is accompanied by a higher risk of falling and fracture [39, 40].

It is important not to forget that vitamin D is a liposoluble vitamin that needs fat from nutrition for adequate resorption. Therefore, we recommend that it is taken with a meal or thereafter to avoid soberness.

#### Calcium supplementation

Calcium is found not only in milk and dairy products. A good basis for a diet with a sufficient supply of calcium for the patients is mineral water rich in calcium, containing approximately 400–500 mg of calcium. This is also possible on a trauma surgery ward. It should be possible to manage about 1000 mg of calcium in combination with a balanced diet containing vegetables, herbs and dairy products such as yoghurt, hard cheese and curds. Of course, there are situations when this is not possible (lactose or other intolerances). Then, an adequate and individual supplementation with calcium is necessary; thus, a total calcium intake of 1000 mg calcium per day is recommended following either supplementation or nutritional intake or a combination thereof. If treated with glucocorticoids, a supplementation with 1000 mg calcium/day is generally recommended.

Patients who have to ingest a proton pump inhibitor (PPI) permanently are a special case. Instead of calcium carbonate, calcium gluconate or calcium citrate is to be administered. For the elderly (>65 years), the long-term intake of a PPI in particular means a higher risk of falling and therefore a higher risk of fracture-associated in-patient treatment [41], decreased trabecular bone mineral density [42], as well as a significantly increased risk of vertebral fractures and hip fractures [43]. Therefore, the indication for PPI treatment and the risk-benefit ratio are to be regularly checked.

#### Specific medical treatment of osteoporosis

Strontium ranelates, SERMs (selective oestrogen receptor modulators: ralixofene, bazedoxifene) and a hormone replacement therapy (HRT) with oestrogens (possibly in combination with a gestagen) are not suitable for use (initiation of specific medical treatment) on a trauma surgical ward due to a poor risk benefit ratio, e.g. risk of thrombosis associated with oral HRT. The recommended drugs for the treatment of osteoporosis are listed in Tables 2 and 3.

**Table 2:** Approval status of selected drugs for specific treatment of osteoporosis

Compound	Post-menopausal women	Men >60 years
Alendronate 70 mg/week	X	–
Alendronate 10 mg/day	X	X
Risedronate 35 mg/week	X	X
Ibandronate oral (150 mg/month) and iv (3 mg/3 months	X	–
Zoledronate iv 5 mg/year	X	X
Denosumab 60 mg sc/6 months	X	X^a^
Teriparatide 20 ug sc/day for max 24 months	X	X

^a^Men with decreased bone density and higher risk of fractures

**Table 3:** Overview of the treatment efficiency of specific osteoporosis treatment for post-menopausal women [4]

Compound	Fewer vertebral body fractures	Fewer peripheral fractures	Fewer proximal femur fractures
Alendronate	A	A	A
Denosumab	A	A	A
Ibandronate	A	B	–
Risedronate	A	A	A
Zoledronate	A	A	A
Teriparatide	A	B	–

#### Oral bisphosphonates

For the intake of oral bisphosphonate patients have to be able to sit up straight for 30 min, renal function has to be sufficiently good (GFR >30 ml/min) and the expected compliance has to be high.

Before initiating anti-resorptive treatment it is essential to look at the patient’s dental chart or at the condition of the jaws (pressure sores) if the patient has dentures. It is recommended that the dentist be informed about the treatment. Regular visits to the dentist for check-ups are also recommended [44].

If it is decided to initiate anti-resorptive treatment with an oral bisphosphonate during a hospital stay, the combination with a proton pump inhibitor (PPI) such as omeprazole is problematic. The resorption rate in the stomach is already very low to start with and is significantly reduced or made impossible by the administration of a PPI [45]. In view of currently available studies, we in this case recommend risedronate 35 mg/week [46, 47].

It is possible to administer 150 mg p.o. of the oral bisphosphonate ibandronate once a month.

The intake frequency must also be considered in terms of compliance; the lower the intake frequency, the better the compliance tends to be [48].

#### IV-bisphosphonate

Bisphosphonates that are injected intravenously may be used when the enteral administration of bisphosphonates is not possible (see above). This requires a GFR >30 ml/min.

Zoledronate (5 mg/year) and ibandronate (3 mg/3 months) are available for this. Zoledronate has been proved to reduce the fracture rate and mortality significantly [49]. This is only true, however, after a time interval of 2 weeks after the operation of a proximal femoral fracture. At this point, patients with an uneventful postoperative course are usually no longer on a trauma surgery ward. From an osteological point of view, it also makes sense to wait with the administration of an iv-bisphosphonate until 6 weeks after a fracture.

#### Teriparatide

Teriparatide (rhPTH 1–34: shortened, recombined form of the human parathormone (1–84)) is an osteoanabolic osteoporosis treatment method that leads to the (re-) augmentation of bone substance and that can reconstruct the micro-architecture of the bone. The osteoanabolic effect (stimulation of proliferation and differentiation of osteoblasts) is achieved by the single application of a small dose of 20 μg teriparatide per day (preferably in the evenings) and, always at the same time, administered subcutaneously by the patients themselves. Contraindications are hypercalcaemia or severe renal insufficiency, Paget’s disease or primary or secondary hyperparathyroidism. Teriparatide is restricted to a maximum treatment duration of 24 months.

Especially from a trauma surgical point of view, the osteoanabolic mechanism of action of teriparatide has on the whole a big advantage compared to anti-resorptive drugs. However, due to its high price and the complex use (daily subcutaneous administration by the patients themselves after a hospital stay), it is not a first choice drug. In a serious course of manifest osteoporosis, teriparatide is definitely to be considered and it has therefore been implemented in our in-patient algorithm.

#### Denosumab

The activity of RANKL on the surface of osteoclasts is specifically inhibited by the high affinity and specificity of denosumab, a fully human, monoclonal antibody of the immunoglobulin-isotype IgG2 that is applied subcutaneously (60 mg/6 months). Denosumab inhibits the binding of RANK-ligands to RANK (surface of osteoblasts) and therefore provides a therapeutic approach in the treatment of osteoporosis and other diseases with loss of bone mass.

It is not necessary to adjust the dose of denosumab for patients suffering from renal dysfunction. Other advantages are the easy subcutaneous administration every 6 months and the lack of febrile reactions in contrast to iv-bisphosphonates. Before the beginning of treatment, the calcium-serum level has to be standard. Patients are to undergo a consistent treatment with calcium and vitamin D. Patients suffering from severe renal dysfunction (Creatine Clearance <30 ml/min) or patients needing dialysis have a higher risk of hypocalcaemia. Severe vitamin D deficiency in particular can predispose patients to get severe symptomatic hypocalcaemia. That is why we recommend checking the level and balancing out the vitamin D deficiency before treatment with denosumab; therefore, an analysis of the calcium level at 7–10 days following treatment with denosumab should be considered; additionally, the cost of denosumab has to be mentioned (Table 4) and considered individually.

**Table 4:** Daily treatment costs of the specific medicinal treatment of osteoporosis

Group	Compound	Dose	€	€ per day
Oral BP	Alendronate	70 mg/week	34.09 (4 tablets)	1.14
	Risedronate	35 mg/week	34.09 (4 tablets)	1.14
	Ibandronate	150 mg/month	34.09	1.14
IV BP	Zoledronate^a^	5 mg/year	548.14	1.50
	Ibandronate	3 mg/3 months	141.42	1.57
SC	Denosumab	60 mg/6 months	311.90	1.73
Osteoanabolic Sc	Teriparatide	20 μg/day pre-filled syringe	625.32 1 month	20.84

Source: Prices from the Rote Liste (German catalogue of drugs) 06-2014; BP ratiopharm©

^a^As there are only generic products with 4 mg of Zoledronate available with different indications for its use, the price of the branded product “Aclasta” was listed

## Results and discussion

Osteoporosis-associated fractures are of increasing importance in trauma surgery. They can be accompanied by significant morbidity and mortality for the patients concerned. In Germany, only a small percentage of patients suffering from osteoporosis receive treatment according to [1, 50]. Algorithms for diagnosing and treating osteoporosis suitable for daily use in traumatology can contribute to improving the deficit in the comprehensive treatment of the disease as well as potentially reduce osteoporosis-associated fractures and avoid failures with regard to the fracture fixation. Based on the current guidelines of the DVO for diagnosing and treating osteoporosis, an algorithm for the diagnosis and treatment of trauma surgical in-patients has been developed. The specific requirements according to DVO guidelines to diagnose osteoporosis are laboratory diagnostics, clinical examinations and conducting a DXA. The differences of our algorithm are that according to the specific requirements for patients, who already gained osteoporotic fractures of the proximal femur or multiple vertebral body fractures I° (Genant) or singular vertebral body fractures II–III° (Genant), the diagnosis osteoporosis can be found with laboratory and radiographic diagnostics and clinical examination. This proceeding allows to start a specific treatment of osteoporosis meanwhile the surgical in-patient treatment. A pilot phase in our clinic showed that training nursing staff how to use the algorithm means significant relief for the doctor in charge. On the basis of the listed inclusion criteria, all the patients were handed the enclosed risk questionnaire. If osteoporosis was suspected, the nursing staff were then able to prepare further diagnostics; DXA and basic laboratory tests were also initiated.

It is therefore possible to diagnose osteoporosis and initiate treatment during the postoperative stay on a trauma surgical ward. For the specific treatment of osteoporosis with the oral bisphosphonate alendronate and risedronate, cost-effective and evidence grade A classified therapeutics are available. IV-bisphosphonates are in principle good alternatives for oral application. When patients are treated with zoledronate (5 mg/year), which is not to be begun earlier than 14 days after the operation, the next institution to provide treatment (e.g. rehab clinic, geriatric ward) has to guarantee cooperation. There are no studies on the time of treatment with denosumab following a fracture. Thus, denosumab can be administered immediately after the operation, taking the present data into account. It can also be used if the patient suffers from renal insufficiency, and it causes no febrile reactions to infusions. That is why we recommend this treatment after checking calcium and 25-OH-vitamin D in patients with contraindications against oral bisphosphonates and/or a severe course of the disease. Direct application during the stay in hospital has to be considered according to the relevant circumstances in the background. If the course of osteoporosis is really severe (e.g. vertebral body fractures during oral anti-resorptive treatment), teriparatide is available as an osteoanabolic substance. Risk, benefit, compliance, and cost all have to be considered individually in all drugs.

It is important to guarantee compliance immediately, in the first year. To this end, not only are the care and healing of fractures to be monitored, but the patients and if necessary their relatives are to be asked about their specific osteoporosis medication, at the latest during the out-patient appointments for postoperative follow-up checks of patients of trauma surgery. Only if the proper drugs are taken or applied can the risk of fracture really be reduced.

Further investigations are required; to complement the lack of a prospective validation of the algorithm, a prospective clinical study is ongoing.

## Conclusion for practice

- Osteoporosis is a frequent underlying disease in elderly patients with fractures following low-energy trauma which is of increasing importance for health economics.
- The doctors in charge have a decisive function when initiating diagnostics and the treatment of osteoporosis to reduce further osteoporosis-associated fractures.
- A treatment algorithm suitable for daily use helps all the surgeons on a trauma surgical ward to diagnose and treat osteoporosis individually.
- The early diagnosis and treatment of osteoporosis can reduce the current deficit in treatment and the associated osteological deuteropathies as well as contribute to allowing trauma surgery patients to maintain their independence.

## Abbreviations

AO/OTA: Arbeitsgemeinschaft für Osteosynthesefragen (AO-Foundation)/Orthopaedic Trauma Association
AP: Alkaline phosphatase
BP: Bisphosphonate
CRP: C-reactive protein
DVO: Dachverband Osteologie = German Osteology Society
DXA: Dual-energy X-ray absorptiometry
ESR: Erythrocyte sedimentation rate
FRAX: Fracture Risk Assessment Tool
GFR: Glomerular filtration rate
HRT: Hormone replacement therapy
IgG2: Immunoglobulin-isotype 2
PPI: Proton pump inhibitor
RANKL: Receptor activator of nuclear factor kappa-B ligand
rhPTH: Recombined form of the human parathormone
SC: Subcutaneous
SERMs: Selective oestrogen receptor modulators
TSH: Thyroid-stimulating hormone
WHO: World Health Organisation
